# Telomere length dynamics in response to DNA damage in malaria parasites

**DOI:** 10.1016/j.isci.2021.102082

**Published:** 2021-01-20

**Authors:** Jake Reed, Laura A. Kirkman, Björn F. Kafsack, Christopher E. Mason, Kirk W. Deitsch

**Affiliations:** 1 Department of Microbiology and Immunology, Weill Cornell Medical College, New York, NY, USA; 2 Department of Physiology and Biophysics, Weill Cornell Medical College, New York, NY, USA; 3 Jill Roberts Center for Inflammatory Bowel Disease, Weill Cornell Medical College, New York, NY, USA; 4 HRH Prince Alwaleed Bin Talal Bin Abdulaziz Alsaud Institute for Computational Biomedicine, Weill Cornell Medical College, New York, NY, USA; 5 Feil Family Brain and Mind Research Institute, Weill Cornell Medical College, New York, NY, USA; 6 WorldQuant Initiative for Quantitative Prediction, Weill Cornell Medical College, New York, NY, USA; 7 Department of Internal Medicine, Division of Infectious Diseases, Weill Cornell Medical College, New York, NY, USA

**Keywords:** astrobiology, chromosome organization, omics, parasitology

## Abstract

Malaria remains a major cause of morbidity and mortality in the developing world. Recent work has implicated chromosome end stability and the repair of DNA breaks through telomere healing as potent drivers of variant antigen diversification, thus associating basic mechanisms for maintaining genome integrity with aspects of host-parasite interactions. Here we applied long-read sequencing technology to precisely examine the dynamics of telomere addition and chromosome end stabilization in response to double-strand breaks within subtelomeric regions. We observed that the process of telomere healing induces the initial synthesis of telomere repeats well in excess of the minimal number required for end stability. However, once stabilized, these newly created telomeres appear to function normally, eventually returning to a length nearing that of intact chromosome ends. These results parallel recent observations in humans, suggesting an evolutionarily conserved mechanism for chromosome end repair.

## Highlights

• Subtelomeric domains of malaria parasites contain virulence genes
• Exposure to DNA damage causes a genome-wide increase in telomere lengths
• Broken chromosome ends undergo telomere healing, extending their length
• The extended number of repeats at healed telomeres lessens over time

## Graphical Abstract

**Figure:**
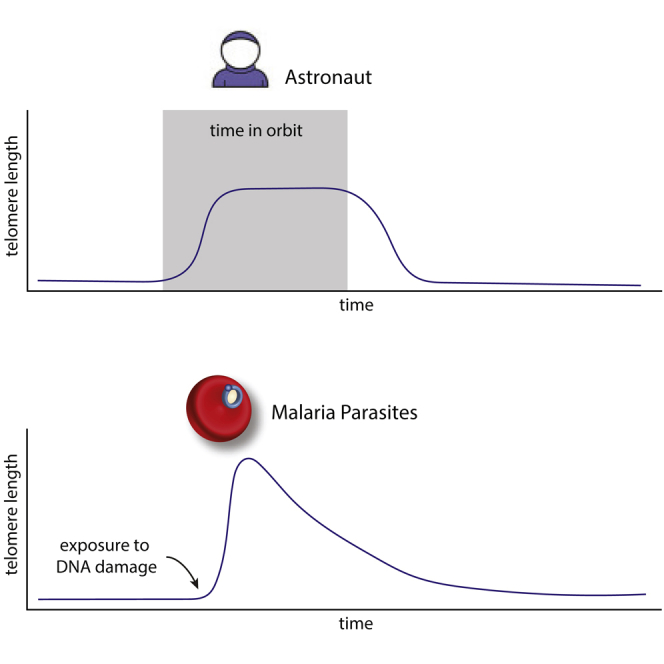

## Introduction

Globally, malaria continues to represent a major threat to public health, infecting over 200 million people a year and causing roughly half a million deaths, the majority being children younger than 5 years (WHO, 2018). The disease is caused by infection with one of several species of eukaryotic parasites of the genus *Plasmodium*, of which *Plasmodium falciparum* is responsible for the most severe forms of the disease. These parasites infect the circulating red blood cells of their mammalian hosts, resulting in severe anemia, inflammatory problems, and disrupted circulation (Miller et al., 2002). The complete genome sequence of *P. falciparum* has now been assembled for numerous geographical isolates, providing information into aspects of virulence, mechanisms of drug resistance, and the plasticity of the parasite's genome (Otto et al., 2018, 2019). In addition, malaria parasites are quite distant from the highly studied model eukaryotes, providing insights into the evolution of many basic molecular processes.

*P. falciparum* has a well-defined chromosome structure that differs somewhat from model eukaryotes. The genome includes 14 chromosomes, and each can be sub-divided into two structural components, the core chromosome containing the centromere and housekeeping genes and the subtelomeric regions (30–120 kb) containing telomere-associated repetitive elements (TAREs), multi-copy gene families encoding variant surface antigens (including *var*, *rifin*, *stevor,* and *Pfmc-2TM*), and telomere repeats (Gardner et al., 2002). These elements are found in a relatively uniform arrangement, with the multi-copy gene families positioned at the boundary of the core genome followed by TAREs and terminating with telomeres at the chromosome ends (Otto et al., 2018). This arrangement functionally partitions the genome into the core, which displays near-complete synteny and a very high degree of sequence conservation when comparing different geographical isolates, and the hyper-variable, multi-copy gene families that are subject to rapid diversification through frequent ectopic recombination events (Bopp et al., 2013; Claessens et al., 2014; Freitas-Junior et al., 2000; Otto et al., 2018). The TAREs are composed of repeat elements that range in size from 21 to 164 bp (Figueiredo et al., 2000). Their function has not been defined, although they are known to transcribe long noncoding RNAs (Broadbent et al., 2011; Sierra-Miranda et al., 2012) and have been hypothesized to play a role in the subnuclear positioning of the chromosome ends, thus possibly contributing to subtelomeric recombination events (Gardner et al., 2002).

The hyper-recombinogenic properties of the subtelomeric regions containing the multi-copy gene families is key to both the parasite's survival and the virulence of malaria caused by *P. falciparum*. Of the various gene families found within these regions, *var* is the best studied. This gene family is highly dynamic, varying in number from 45 to 90 genes within the haploid genomes of different parasite isolates (Otto et al., 2018). Each *var* gene encodes a different form of the surface antigen *Plasmodium falciparum* erythrocyte protein 1 (PfEMP1), which is expressed on the surface of the infected red blood cell and is responsible for parasite sequestration away from the spleen via adhesion to the vascular endothelium. Sequestration within blood vessels is not only one of the main mechanisms for immune evasion but also directly implicated in a number of malaria disease pathologies including cerebral malaria and placental malaria. Importantly, the position of PfEMP1 on the infected cell surface exposes it to the humoral immune system of its host, and infected individuals readily make antibodies that efficiently recognize and destroy infected cells (Scherf et al., 2008). Thus, to avoid clearance by the antibody response, parasites alternate which *var* gene is expressed, effectively cycling through their repertoire of genes in a process that is regulated epigenetically (Deitsch and Dzikowski, 2017). This process is referred to as antigenic variation and is dependent on extensive variability between *var* genes to avoid cross-reactive antibody responses to different forms of PfEMP1. Moreover, to avoid pre-existing immunity from previous infections, different parasites circulating within a geographical area must differ substantially from each other, thus providing a strong selection pressure for continuous and rapid *var* gene diversification.

To explore the extent of *var* gene diversity globally, Otto et al. analyzed 714 clinical malaria isolates across 12 countries (Otto et al., 2018, 2019). They found that the isolates in each country contained between 6 and 21 shared *var* genes based on sequence homology, indicating that the vast majority (79%–94%) of *var* genes in each region are unique (Otto et al., 2019). Previous geographical surveys of *var* gene sequences detected a similar degree of diversity (Barry et al., 2007; Chen et al., 2011), indicating that the hyper-recombinogenic properties of the subtelomeric regions are a universal property of *P. falciparum* parasites. The structure of the subtelomeric regions, their positioning within clusters located at the nuclear periphery, and the molecular processes of telomere healing, homologous recombination, and telomere maintenance have all been implicated in the multi-copy gene family diversification process. For example, Zhang et al. recently reported that a single double-strand break (DSB) within a sub-telomeric region can lead to a cascade of recombination between *var* genes on different chromosomes, leading to the creation of new chimeric *var* genes through a combination of telomere healing and homologous recombination (Zhang et al., 2019). This process was also noted in three other clones in previously published data (Claessens et al., 2014; Zhang et al., 2019), suggesting that this is a common mechanism leading to the diversification of this highly variant gene family. Thus the rapid generation of antigen diversity appears to be inherently linked to the maintenance of chromosome ends.

The primary function of telomeres is to prevent degradation of genetic material during replication, often described as the end-replication problem (Saretzki, 2018). The telomeres of eukaryotic organisms consist of tandem arrays of short repeat sequences incorporated at the chromosome ends by telomerase. This enzymatic activity enables replicating cells to counter the shortening of telomeres during DNA replication, thereby maintaining the stability and integrity of the chromosome ends. In addition, in the event of a chromosome break within a subtelomeric region, telomerase can stabilize the broken chromosome end through *de novo* telomere addition (also called telomere healing), a process recently linked to accelerated variant antigen diversification in *P. falciparum* (Zhang et al., 2019). Given that destabilization of chromosome ends can lead to recombinational cascades that result in rapid diversification of variant antigen genes (Claessens et al., 2014; Freitas-Junior et al., 2000), and that telomere healing appears to play a key role in this process (Calhoun et al., 2017; Zhang et al., 2019), the unusual nature of *Plasmodium* telomere maintenance has acquired renewed attention (Arnot, 2019). Such studies could provide insights into how malaria parasites balance the need to maintain genome integrity while also undergoing rapid genetic diversification. Here we used long-read sequencing technology to investigate telomere dynamics in *P. falciparum* and to determine how the DNA damage response influences these dynamics.

## Results

### SMRT sequencing allows for accurate length determination of *Plasmodium falciparum* telomeres

*P. falciparum* telomeres are highly variable in length, ranging in size from 0.5 to 6.5 kb (Figueiredo et al., 2002) and are composed of a 7-bp repeat, which varies between the sequence 5′-TTCAGGG-3' and 5′-TTTAGGG-3' (Mattei and Scherf, 1994). Given that telomeres are continuously turning over as part of chromosome replication, their length can vary depending on telomerase activity and recruitment of the enzyme to specific chromosome ends. The consequences of differing telomere lengths are not well understood, although recent work has shown that environmental conditions can influence telomere length distribution (Garrett-Bakelman et al., 2019). In the past, telomere lengths have been estimated by pulse field gel electrophoresis (Biggs et al., 1989; Dore et al., 1990; Ponzi et al., 1990), quantitative real-time polymerase chain reaction (Borresen et al., 2016; Cawthon, 2009), or *in situ* hybridization (Lansdorp et al., 1996; Sishc et al., 2015), with each method providing an estimate of the number of repeats found at the chromosome ends. However, new long-read sequencing methods, called SMRT (single-molecule real-time) sequencing (Chin et al., 2013), are capable of extending through complete arrays of telomere repeats, thus potentially providing a new method to directly measure telomere lengths at each individual chromosome end, and to detect changes in telomere length caused by alterations in environmental conditions. We explored the utility of this method by applying it to the study of telomere dynamics in *P. falciparum*.

**Figure 1:**
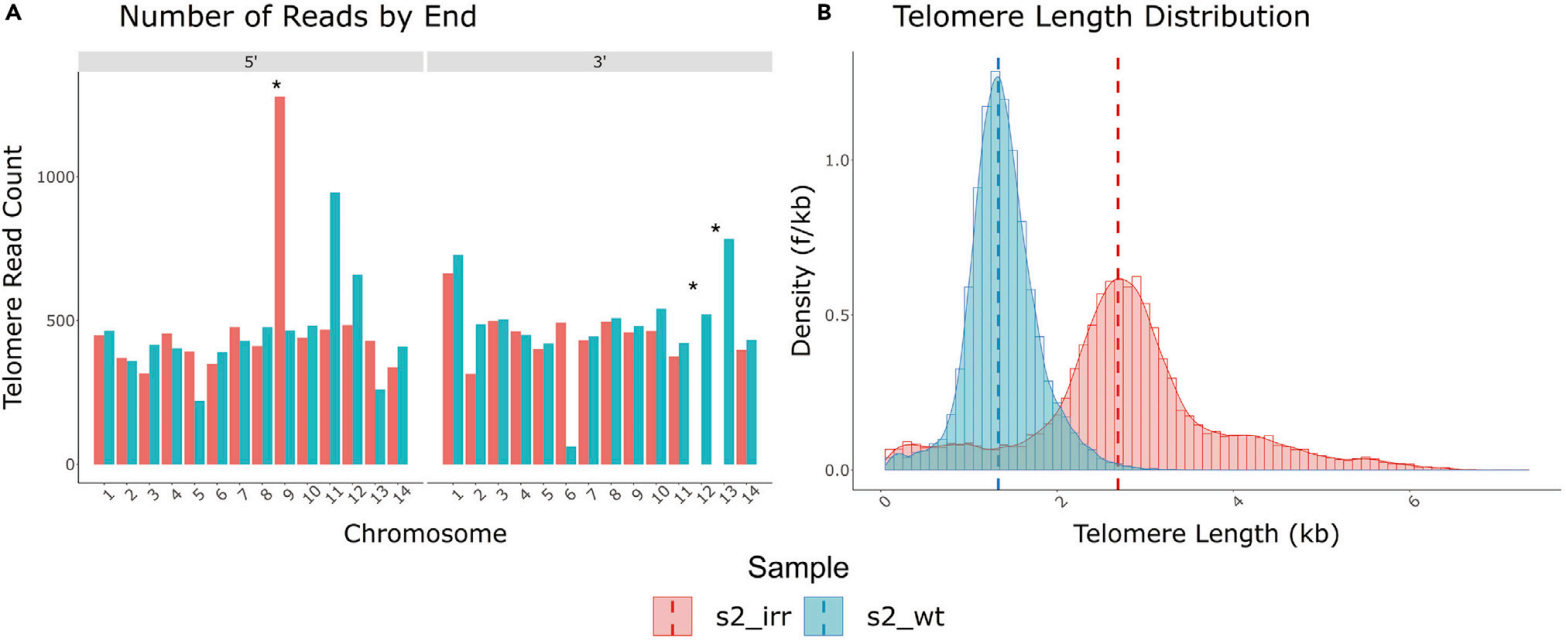
Total telomere length distributions in irradiated and wild-type parasites (A) Number of telomere reads assigned to each chromosome end by radiation status, irradiated (red) or wild-type (blue). (B) Comparison of telomere length distributions of the irradiated (red) and wild-type (blue) samples. The red and blue dashed lines indicate the modes of the irradiated and wild-type samples, respectively. ∗ Designates a duplication of the 5′ end of chromosome 9 onto the 3′ ends of chromosomes 12 and 13 (Calhoun et al., 2017).

In a recent study of DNA repair in response to DNA damage, we applied SMRT sequencing technology to generate *de novo* sequence assemblies from cultured parasites (Calhoun et al., 2017); however, the focus of that analysis was on structural changes and telomere length was not examined. In the current study, we resequenced the same parasite lines to better resolve the genome and investigate telomere length dynamics. Two independent parasite lines, one grown under normal culture conditions and the other exposed to DNA damage through irradiation, were studied. Parasite DNA was isolated using phenol-chloroform extraction (Swamy et al., 2011) and sequenced using SMRT technology from Pacific Biosciences (PacBio). PacBio was chosen as the sequencing platform because it produces reads in the tens of kilobase range as opposed to 50–250 base pair range as is typical for most next-generation sequencing platforms. This allows for greater resolution of telomere tracts. A custom Bash (Free Software Foundation, 2007) script utilizing Hypergeometric Optimization of Motif EnRichment (HOMER) (Heinz et al., 2010) was used in combination with the most current GenBank (Sayers et al., 2019) reference genome for *Plasmodium falciparum* 3D7 to determine telomere repeat motifs, which was used to pull down all reads with the discovered telomere motifs. Reads that contained the telomere motifs were then analyzed for telomere repeat content and assigned a percent telomere content based on a 200-bp rolling window with a 100-bp step. Quality control, length assessment, and chromosome end assignment were performed using a custom R (Team, 2013) script and minimap2 (Li, 2018). Of the 28,506 reads containing the canonical *P. falciparum* telomere repeat motifs, we were able to assign 25,280 (88.7 %) reads to their respective chromosome end. Of the 56 predicted telomere tracts from the two samples, we were able to confidently reconstruct 54 unique telomere tracts with a mean coverage depth of 468 ± 23.4 (Figure 1A). As expected, the 3′ ends of chromosomes 12 and 13 were absent in the irradiated parasites. This is due to a previously documented event where the 5′ end of chromosome 9 was duplicated onto the 3′ end of chromosomes 12 and 13 (Figure 1A). The evidence for this event is discussed in detail in a previous publication (Calhoun et al., 2017).

To determine telomere length, a custom R (Team, 2013) script was used to first determine appropriate start and end thresholds based on a sample of 1,000 reads. Then the start threshold was used to ensure that only one end of each read contained telomeric repeats, and the end threshold was used to estimate the length based on a 200-bp window falling below a telomere motif content threshold of 42% at which point the number of 200-bp windows were counted. This allowed us to resolve telomere length to within 100 bp on a per read basis. The telomere length distribution was then plotted, and the modes were determined using the modes R package (Team, 2013). Thus, our ability to use long-read sequencing technology combined with a computational approach that measured telomere length at 100 bp resolution per read allowed us to confidently determine telomere length distributions in *P. falciparum*.

### Irradiated malaria parasites have longer telomeres

In our previous study, we examined DNA repair within subtelomeric regions of *P. falciparum* using X-ray irradiation as a source of DNA damage (Calhoun et al., 2017). This study identified both telomere healing and homologous recombination as important mechanisms for maintaining genome integrity in response to DNA DSBs; however, how these repair events influenced telomere length was not examined. Given the repair events that we previously described frequently involved telomere healing, it was clear that telomerase activity was likely influencing the repair process. This led us to investigate if such mechanisms of repair might alter the telomere lengths observed in these parasites. We therefore more precisely examined dynamics specifically at the chromosome ends by directly comparing telomere length distributions in genomic DNA extracted from both the irradiated and non-irradiated parasite cultures.

For the irradiated samples, mixed stage parasites were exposed to X-rays at 100 Gy for three iterations, allowing the parasites to regrow in between each exposure (Calhoun et al., 2017). After recovery, DNA was isolated from a clonal line of parasites obtained from the irradiated population, sequenced using PacBio technology, and analyzed as described earlier. When the telomere lengths derived from both the irradiated and non-irradiated samples are displayed side by side, the telomeres from the irradiated sample displayed a clear increase in average length (p <0.001) (Figure 1B). The mean length for the wild-type parasites was 1.383 kb, whereas that for the irradiated parasites was 2.776 kb, which amounts to a doubling of general telomere length due to exposure to X-rays (Figure 1B).

### Sites of telomere healing have significantly increased numbers of telomere repeats

We were curious if the increased average telomere length observed in the irradiated line was due to a general increase in the number of telomere repeats across all chromosome ends, or if instead specific telomeres displayed disproportionate increases in length. We were especially interested in sites of recent telomere healing where increased recruitment and processivity of telomerase are required for synthesizing a new telomere *de novo*. The unique structure of *P. falciparum* subtelomeric domains makes identifying healing events simple and unambiguous. All wild-type subtelomeric domains include the telomere repeats at the chromosome end flanked by 10–25 kb of TAREs followed by members of the multi-copy variant antigen gene families. The loss of TAREs and/or insertion of telomere repeats within a region containing multi-copy gene family members is a hallmark of telomere healing, with the site of telomere addition easily identifiable as the position where the telomere repeat sequences initiate and the chromosome prematurely terminates. Furthermore, by comparing genome assemblies before and after exposure to radiation, we can identify telomere healing events that were present in the parasite’s genome before exposure to radiation (past events) and recent events resulting from radiation exposure.

Our previous analysis identified six sites of telomere healing that existed in our parasite line before radiation exposure and two sites of healing that were the direct result of exposure to radiation, the 5′ end of chromosome 1 and the 3′ end of chromosome 2 (Calhoun et al., 2017). Our current analysis also identified a third radiation-induced telomere healing event on the 5′ end of chromosome 3 (Figure S1). Furthermore, as the DNA for analysis was isolated from parasites shortly after they had recovered from radiation exposure, we had the unique opportunity to measure telomere lengths at sites of relatively recent telomere healing events. Thus, by comparing the telomere lengths of all 28 chromosome ends for both the irradiated and non-irradiated lines, we could examine telomere dynamics as parasites recovered from DNA damage.

**Figure 2:**
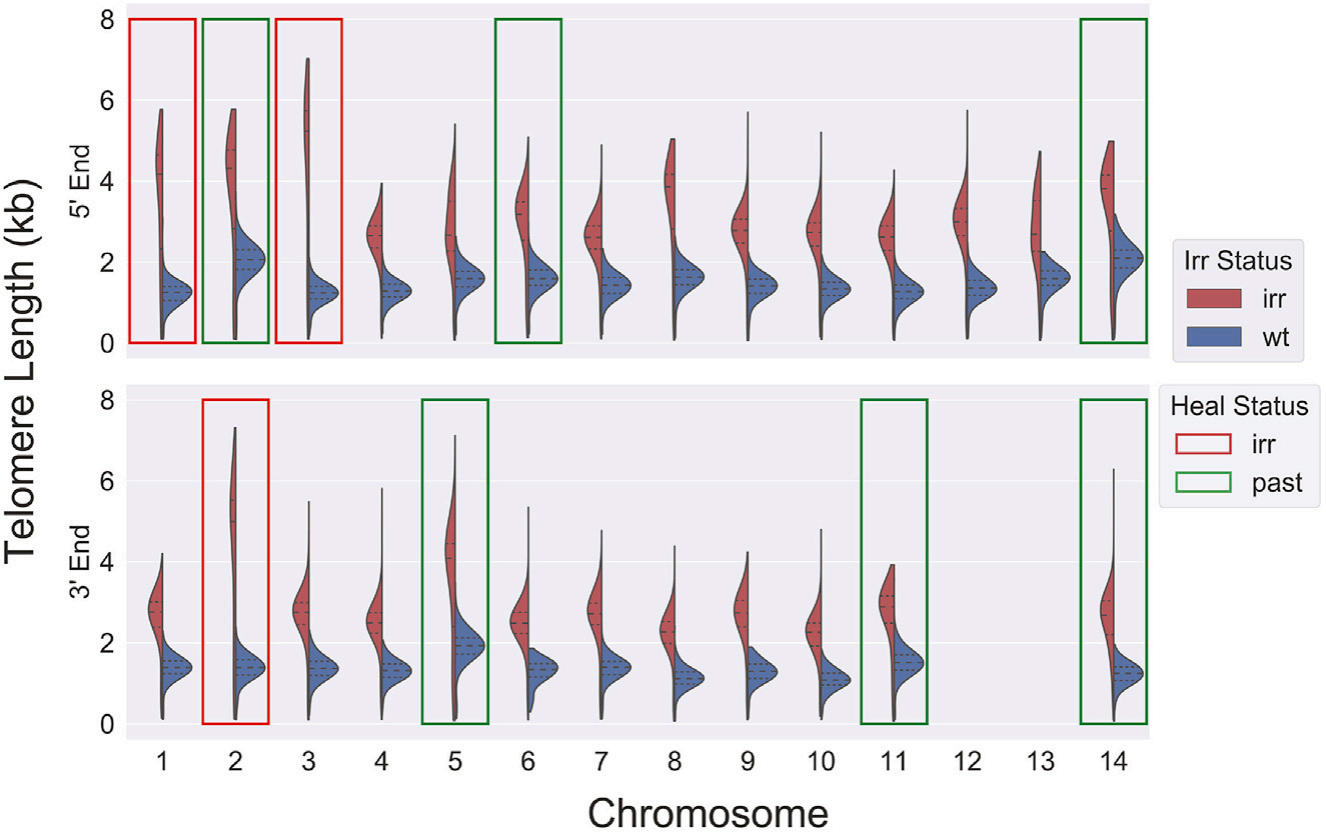
Comparison of telomere lengths by end and radiation status Red boxes indicate telomere healing events resulting from exposure to radiation, whereas green boxes indicate telomere healing events found in the original population, before exposure to radiation.

Individual comparisons of the telomere lengths of each chromosome end detected a general trend toward longer telomeres at all chromosome ends in the irradiated parasite line (Figure 2), with recently healed telomeres displaying particularly pronounced increases in length (Figure 2, red boxes). These trends are also evident in a previous sequencing run on a PacBio RS II instrument, albeit with lower resolution (Figure S2), and greatly increased telomere length due to healing and general telomere lengthening due to radiation is maintained even upon downsampling to 35 reads per chromosome end (Figure S3). This suggests that the DNA damage induced by exposure to radiation led to a general increase in telomerase activity, thus leading to an increase in telomere lengths at all chromosome ends. However, the recruitment of telomerase to sites of telomere healing induces the initial synthesis of telomere repeats well in excess of the minimal number required for end stability, thus leading to the particularly long tracts of telomere repeats observed at healed chromosome ends. This increased length was sufficiently stable that it was easily observed after the several months in culture required for recovery from irradiation and for expansion of the parasite population after cloning.

### Lengthened telomeres at sites of healing shorten over time, but remain extended

**Figure 3:**
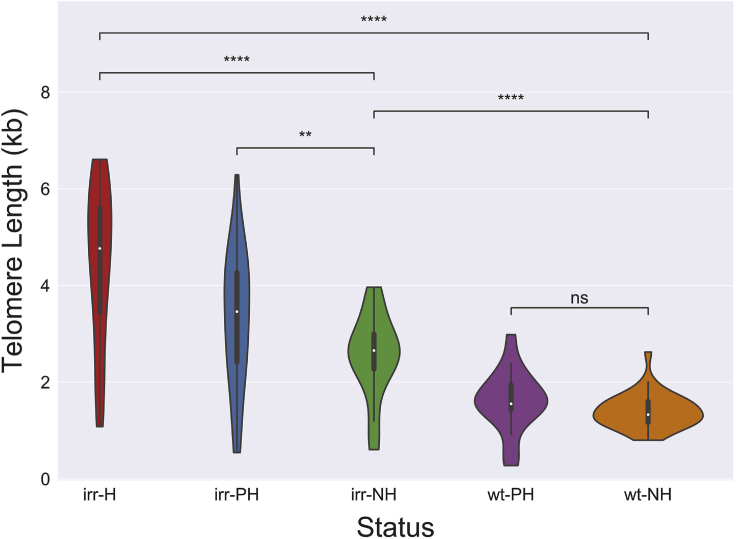
Telomere lengths by radiation and healing status Telomere lengths were assessed after a random downsampling to n = 35. Welch's t test was used to determine significance. The samples are ordered from left to right as irradiated healed telomere tracts (irr-H), irradiated past healed telomere tracts (irr-PH), irradiated non-healed telomere tracts (irr-NH), wild-type past healed telomere tracts (wt-PH), and wild-type non-healed telomere tracts (wt-NH). p value annotation legend: ns: 5.00x10^-2^ < p ≤ 1.00x10; ∗∗1.00x10^-3^ < p ≤ 1.00x10^-2^; ∗∗∗∗p ≤ 1.00x10^-4^.

Our examination of the recently irradiated parasite line indicated that the changes in telomere repeat numbers were relatively stable, at least over the course of the several months required to complete the experiment. This raised the possibility that the increased telomere length at sites of healing might be required to compensate for the altered structure of the subtelomeric domain, for example, due to the loss of TAREs after healing, and therefore permanent. To investigate this possibility, we took advantage of our recent identification of six sites of telomere healing that were present in the original population of parasites before irradiation (Figure 2, green boxes) (Calhoun et al., 2017). These healing events occurred sometime in the history of this parasite line, and five are found in the reference sequence of 3D7 (Eupathdb), indicating they likely occurred before the isolation of the 3D7 clone over 30 years ago. This enabled us to compare chromosome healing events that happened in the distant past with very recent events and thus infer details of telomere length dynamics and stability in malaria parasites. To facilitate this analysis, we grouped telomere tracts into bins based on healing status and whether the parasites had been exposed to radiation and then compared the length differences of each class using Welch’s t test to evaluate the significance of changes (Figure 3).

When telomeres from irradiated parasites were compared with non-irradiated controls, non-healed telomeres (irr-NH) increased in length by 1.146 kb (1.82-fold increase), whereas the healed ends (irr-H) displayed a much greater increase of 3.042 kb (3.18-fold increase) (Figure 3 and Table 1). As described earlier, this is consistent with a general increase in telomerase activity in response to irradiation leading to a lengthening of all chromosome ends, with an even greater increase in telomerase activity during *de novo* telomere addition. The significance of these events by Welch’s t test was p < 0.001 after downsampling to 35 reads per bin (Figure 3).

**Table 1:** Telomere length increases

Status (Rad-Healing)	Percentiles (kb)			Mean ± SE (kb)	Fold Increase
	25th	50th	75th		
**n = 35**					

irr-H	3.434	4.766	5.603	4.440 ± 0.271	3.18
irr-PH	2.416	3.460	4.268	3.320 ± 0.232	2.37
irr-NH	2.269	2.654	2.995	2.544 ± 0.142	1.82
wt-PH	1.434	1.553	1.943	1.605 ± 0.104	1.15
wt-NH	1.169	1.330	1.601	1.398 ± 0.060	0.00

**n = 1,075**					

irr-H	2.352	4.432	5.332	3.822 ± 0.056	2.90
irr-PH	2.425	3.128	3.997	3.041 ± 0.038	2.31
irr-NH	2.271	2.662	2.971	2.571 ± 0.024	1.95
wt-PH	1.376	1.710	2.054	1.693 ± 0.016	1.28
wt-NH	1.125	1.330	1.521	1.318 ± 0.010	0.00

The samples are grouped in the left column by irradiation status (wt: wild-type; irr: irradiated) and healing status (H: recently healed due to irradiation; PH: pre-radiation healing; NH: not healed).

To investigate what happens to healed telomeres over time, we used data from the non-irradiated parasites to compare telomere repeat lengths at sites of past telomere healing events with non-healed telomeres (wt-PH versus wt-NH). This also revealed a modest, non-significant increase in telomere length of 207 bp, suggesting that the increased telomere length due to healing eventually returns to a length approaching that of non-healed chromosome ends. Interestingly, a similar comparison of previously healed and non-healed telomeres from the irradiated parasites (irr-PH versus irr-NH, Figure 3) indicated that these lengths are significantly different, suggesting the possibility that exposure to radiation leads to the increase of all telomeres to similar lengths (previously healed or non-healed), but that the healed telomeres remain somewhat longer after recovering to a “normal” set point (Figure 3).

**Figure 4:**
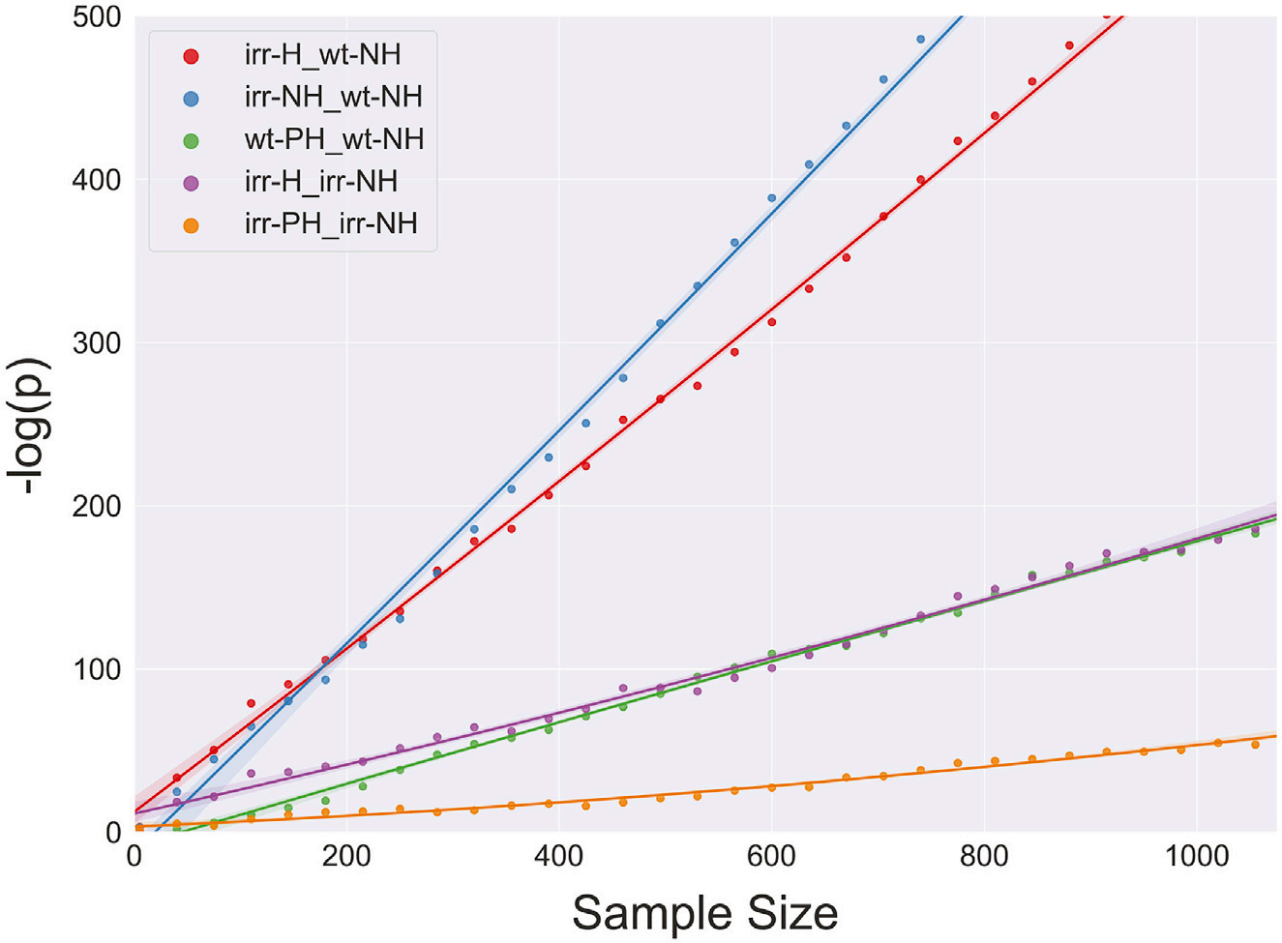
Sample size impact on p value Sample size was randomly downsampled from the minimum number of reads per grouping (1,075 reads in the irradiated healed set). p Values were generated based on different grouping comparisons while sample size was progressively increased from *n* = 35. The p values generated approached zero in an exponential fashion, therefore -log(p) was plotted to make differences discernable. Irradiated healed telomeres versus wild-type non-healed (irr-H_wt-NH) (red) and irradiated non-healed telomeres versus wild-type non-healed telomeres (irr-NH_wt-NH) (blue) comparisons consistently displayed the most significant differences in telomere length. Wild-type past healed telomeres versus wild-type non-healed telomeres (wt-PH_wt-NH) (green) and irradiated healed telomeres versus irradiated non-healed telomeres (irr-H_irr-NH) (purple) displayed marginally significant telomere length differences, and the length difference between irradiated past healed telomeres and irradiated non-healed telomeres (irr-PH_irr-NH) (orange) was non-significant initially but became more significant as the sample size increased.

The trends described earlier are evident across all sample sizes (35–1,075), indicating consistent differences in this data set (Figure 4). Across all sample sizes, the most significant length differences are between the irr-H versus wt-NH and irr-NH versus wt-NH comparisons whose p values continue to separate further from the other comparisons as sample number increases, indicating that there are consistent and highly significant increases in lengthening of telomeres due to healing and exposure to radiation (Figure 4).

## Discussion

Like in all eukaryotic organisms, telomeres are required for maintaining chromosome stability and genome integrity in malaria parasites. However, in these parasites they also contribute to a unique subtelomeric structure that includes the primary antigenic and virulence determinants of malaria caused by *P. falciparum*. Moreover, telomere healing was recently shown to be a primary contributor to the recombination process that drives the sequence diversification of subtelomeric variant antigen-encoding genes (Zhang et al., 2019), thus the synthesis and stability of telomeres directly contributes to host-parasite interactions and the virulence of the disease. Telomere healing has been found in *P. falciparum* field isolates (Biggs et al., 1989; Dharia et al., 2010; Murillo et al., 2015), indicating that this mechanism of chromosome stabilization occurs frequently in a natural setting and is not just a phenomenon observed in laboratory-cultured parasites. In fact, a common telomere healing event that leads to loss of a gene encoding a protein used in rapid diagnostic tests was recently found to be responsible for decreased efficacy of clinical diagnosis, reinforcing the importance of this process (Gamboa et al., 2010; Maltha et al., 2012). Therefore understanding the enzymatic activities underlying telomere synthesis and maintenance will provide insights into an important biological process of malaria parasites.

The role of telomere healing in driving *var* gene diversification was recently described by Zhang et al. (2019). Their model proposes that when a DSB occurs within a subtelomeric region, repair of the break by homologous recombination competes with telomere healing to stabilize the chromosome end. When healing occurs, this liberates a hyper-recombinogenic free DNA fragment that often contains *var* genes. This fragment can initiate a cascade of recombination events leading to the generation of new chimeric *var* genes, thus increasing antigen diversity. Increased telomerase activity, for example, as observed here after exposure to radiation, would presumably skew such repair events toward telomere healing and thus accelerate the diversification process. Therefore the basic biology of telomere maintenance and chromosome end repair in *P. falciparum* plays a direct role in pathogenesis and immune evasion.

SMRT sequencing provides a new method for assaying changes in telomere length with remarkable precision. In addition, as opposed to some other methods, SMRT sequencing also enables easy detection of telomere lengths at individual chromosome ends, thereby allowing the detection of changes that occur only at specific regions of the genome. This enabled us to directly measure the number of repeats resulting from a specific telomere healing event, something that was not previously possible. Using this unique approach, we were able to determine that telomere healing events in *P. falciparum* lead to initial over-lengthening of the telomere, resulting in disproportionately long stretches of telomere repeats at sites of healing. We were also able to determine that the increased length is not permanent and that the extended telomeres appear to eventually return to near baseline. This parallels recent observations in humans in which extended occupation of the international space station led to a temporary increase in telomere length in nucleated blood cells (Garrett-Bakelman et al., 2019). The telomere lengths returned to baseline after the subject returned to earth. Although the cause of the increased telomere length cannot be known for certain, it is tempting to speculate that exposure to increased radiation while in space led to telomere lengthening, similar to what we observed after irradiation of malaria parasites. This suggests a shared response to DNA damage and repair of broken chromosome ends that is conserved across the broad evolutionary distance that separates humans from protozoan parasites.

SMRT sequencing is becoming much more commonly used for the assembly of full genome sequences in a variety of organisms. Given that the sequencing reads included in all these datasets likely include the telomeres, it should be possible to derive telomere length assessments directly using the methods described here without additional experimentation. Thus, ever-increasing application of SMRT sequencing is likely to provide an influx of new datasets with valuable information for researchers interested in the dynamics of telomere maintenance under a variety of conditions. For malaria parasites, our analysis provides additional insights into the unique structure of the chromosome ends and how they are maintained. Given the importance of telomeres and chromosome end stability to variant antigen diversity and expression, the data and methods presented here add to our increasing understanding of this important aspect of *Plasmodium* biology.

### Limitations of the study

This study employed exposure to radiation as the source of DNA damage, which led to an increase in the number of telomere repeats at each chromosome end. However, in a natural setting, radiation is unlikely to be a significant source of increased DNA damage. Some alternative sources that could be active during an infection include products of the host immune response or various antimalarial drugs, including artemisinin. The study focused on telomere lengths of parasite populations that have been cultured *in vitro* for decades. It would be interesting to investigate natural telomere lengths in parasites recently obtained directly from the field.

### Resource availability

#### Lead contact

Further information and requests for resources and reagents should be directed to and will be fulfilled by the Lead Contact, Christopher Mason (chm2042@med.cornell.edu).

#### Materials availability

Parasite lines and genomic DNA samples are available from the authors upon request without restriction.

#### Data and code availability

The genomic DNA sequence data have been deposited in an SRA here: https://www.ncbi.nlm.nih.gov/bioproject/PRJNA677982. All scripts used in the analysis of these data are available on github (https://github.com/jake-bioinfo/tld).

## Methods

All methods can be found in the accompanying Transparent methods supplemental file.

## Supplemental information

Document S1. Transparent methods, Figures S1–S3, and Table S1
