# Supplementary material for: Telomere length dynamics in response to DNA damage in malaria parasites
Source: iScience. 2021 Jan 20;24(2):102082. doi: 10.1016/j.isci.2021.102082 (PMC7887396; doi:10.1016/j.isci.2021.102082)
Supplement: Document S1. Transparent methods, Figures S1–S3, and Table S1 [file mmc1.pdf]

iScience, Volume *24*

Supplemental Information

Telomere length dynamics

in response to DNA damage

in malaria parasites

Jake Reed, Laura A. Kirkman, Björn F. Kafsack, Christopher E. Mason, and Kirk W. Deitsch

SUPPLEMENTAL INFORMATION

Figure S1. Pile-up a 200kb region demonstrating the truncation on the end of chromosome 3, related to Figure 2

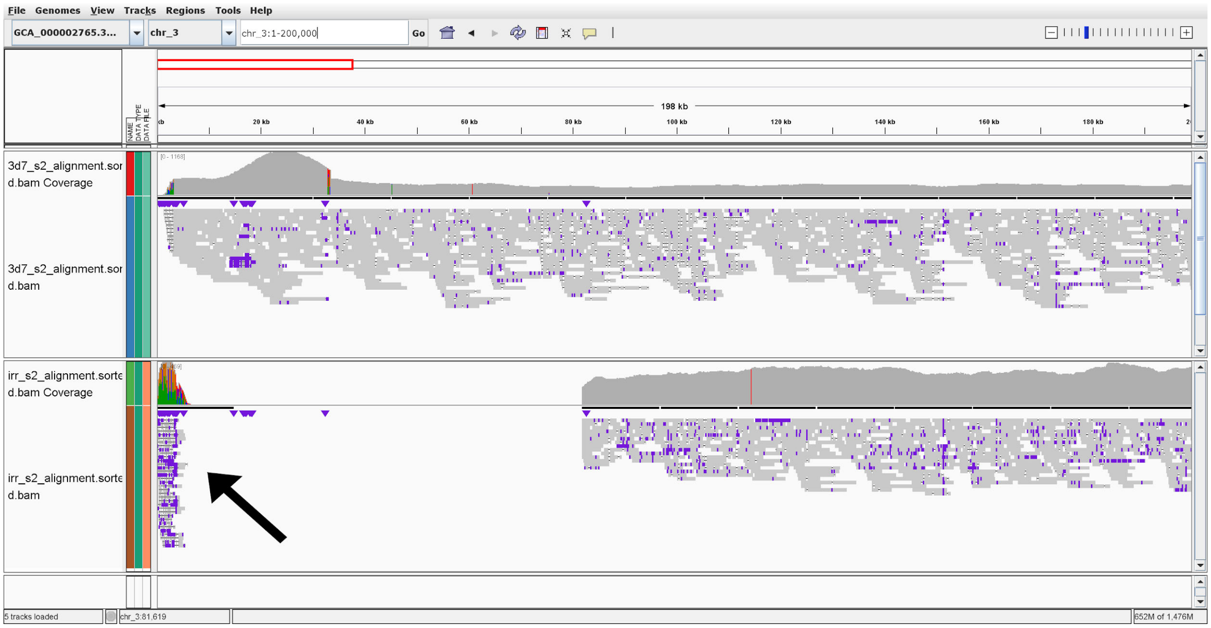

Supplemental Figure 1: Pile-up a 200kb region demonstrating the truncation on the end of chromosome 3. Circular consensus sequences reads were aligned to the reference genome using minimap 2 (Li, 2018). The wild-type parasite reads are on the upper panel while the irradiated parasite reads are on the lower panel. The truncation of the irradiated parasites is evident on the bottom panel. The truncation led to an 82kb deletion on the 5' or "left" end of chromosome 3. The sequence reads from the irradiated parasite include the incorporation of telomere repeat sequences at the site of the deletion, verifying that a telomere healing event has stabilized the chromosome end. This can be observed in the pile up as reads mapping to the extreme left end of the reference sequence, where telomere repeat sequences reside (arrow).

Figure S2. Previous Sequencing Data Results, related to Figure 2.

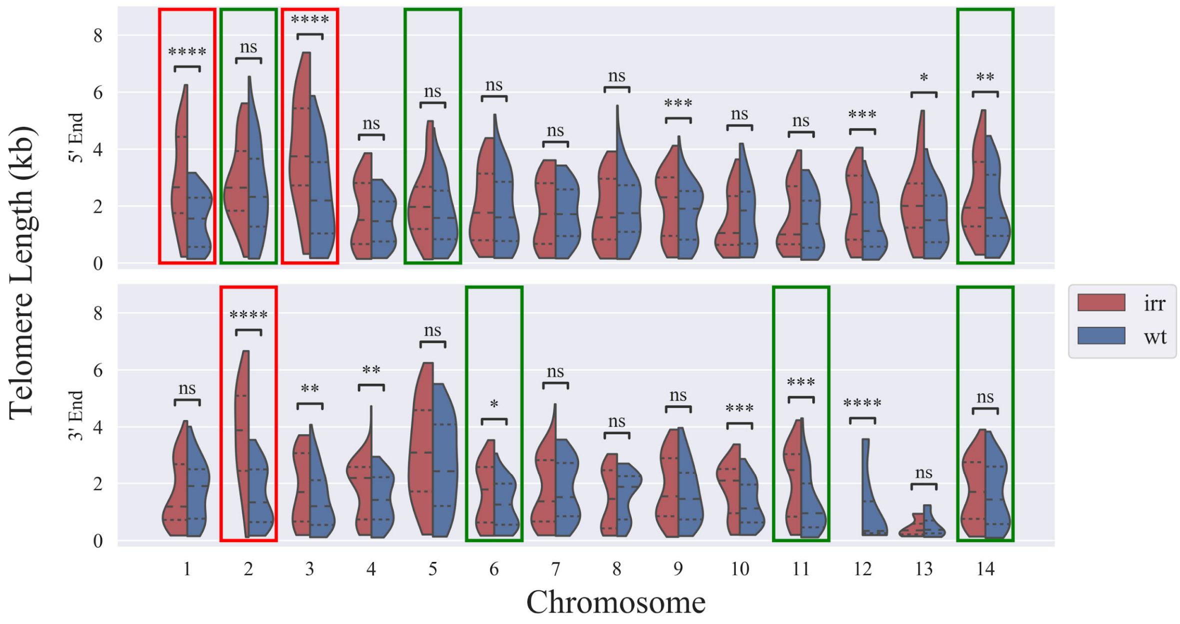

Supplemental Figure 2: Previous sequencing data results. Data generated from PacBio RS II instrument with the same clones show very similar results. Truncated telomeres (“left” end of chromosomes 1 and 3 and “right” end of chromosome 2) are significantly longer by the Welch’s two sided t-test, while in general, radiation causes a lengthening of all telomeres.

Figure S3. Telomere Lengths by Chromosome End Downsampled to 35 Reads per End, related to Figure 2.

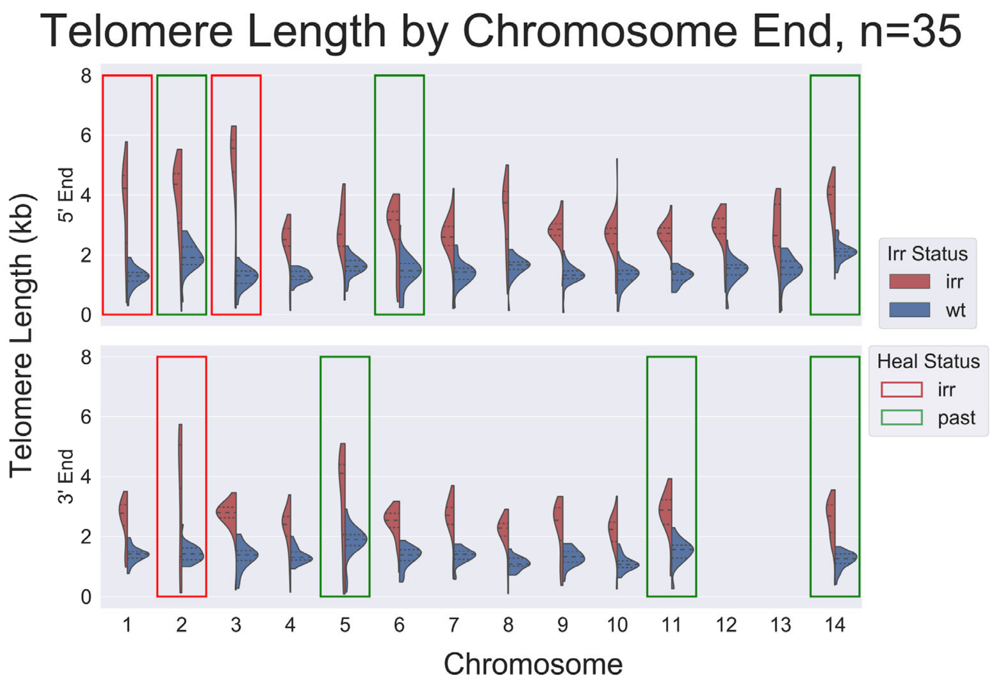

Supplemental Figure 3: Telomere lengths by chromosome end downsampled to 35 reads per end. Telomere lengths after randomly downsampling to 35 reads per chromosome end show that truncated telomeres are lengthened the most while radiation in general increases telomere length. The past healed ends are also closer in length to the irradiated ends than to the irradiated healed ends. This shows that there is no bias based on sampling due to the irradiated sample having been sequenced to a higher depth.

Table S1: Basic Sequencing Statistics, related to Figures 1, 2 and 3

	C3 (non-irradiated)	E8 (irradiated)
Polymerase Read Bases	176,356,836,133	267,554,651,705
Polymerase Reads	3,033,878	4,986,496
Polymerase Read Mean	58,129	53,656
Polymerase Read N50	182,803	140,678
Subread Length (mean)	7,320	6,654
Subread N50	8,491	7,638
Insert Length (mean)	12,978	14,495
Insert N50	26,672	28,152
Unique Molecular Yield	35,684,548,608	64,645,750,784

Supplemental Table 1: Basic sequencing statistics. The telomere read lengths were normalized by the subread N50 read length based on the PacBio Sequel 2 statistics. The basic sequencing stats show that the sequencing runs for the non-irradiated and irradiated runs are very similar with regards to the majority of statistics, excepting throughput. The margin of difference for all other stats is < 25% difference (Polymerase Read N50) and most have a < 10% difference (Polymerase Read Mean, Subread Length Mean, Subread N50, Insert Length mean, and Insert N50).

TRANSPARENT METHODS

Culturing and irradiation.

The method for parasite culturing is based on the original method described by Trager and Jensen with modifications as described in Calhoun et al. (Calhoun et al., 2017). Briefly, *P. falciparum* was cultured in RPMI complete media supplemented with Albumax II and gentamicin. The cultures were maintained in a 90% nitrogen, 5% carbon dioxide, 5% oxygen atmosphere at 37° Celsius. The ionizing radiation dose was 100-Gy, and mixed-stage parasites were consecutively irradiated three times. The cultures were allowed to recover to normal growth levels between each radiation exposure. Deletion of *var* genes was assayed by quantitative polymerase chain reaction (PCR)

with genomic DNA (gDNA) using a PCR *var* panel developed by Salanti and colleagues (Salanti et al., 2003). The whole-genome analysis was performed on a subclone chosen due to the loss of three subtelomeric *var* clusters based on the previously described assay.

Genomic DNA isolation and PacBio Library Preparation.

This technique was previously described in Vembar et al (Vembar et al., 2016). Parasite gDNA was isolated from cultures at 5-8% parasitemia. The DNA extraction method used was phenol-chloroform and ethanol precipitation. Library preparation, size selection and sequencing carried out at Cold Spring Harbor Laboratories. Briefly, SMRTbell template prep kit 1.0 (Pacific Biosciences) was used for the library preparation along with size selection using BluePippin for 20kb fragments which was then sequenced on the PacBio Sequel II platform. Each sample pool was barcoded (Sequel_16_barcodes_v3), pooled for equimolar mixing, and then sequenced using a single SMRT cell. The controls (LID50609-Cell3) yielded 3,033,878 polymerase reads with a mean read length of 58,129 nt and a subread N50 of 8,491 nt, whereas the irradiated samples (LID50610-Cells1) yielded greater total reads, with a total of 4,986,496 reads, a mean read length of 53,656 nt, and a subread N500 of 7,638 nt. These methods are described in more detail in Calhoun et al (Calhoun et al., 2017). The genomic DNA samples analysed for this study were originally isolated and described by Calhoun et al (Calhoun et al., 2017) and resequenced for the current analysis.

Extracting telomere reads

The computation method utilized was a custom script written in Bash (Foundation, 2007). It involved pattern recognition based on the previously published *P. falciparum* telomere motifs (Mattei and Scherf, 1994). The sequencing runs for the two samples yielded similar results; however, the throughput for the irradiated sample was 1.52X the non-irradiated while the other sequencing metrics were only marginally different [Table S1]. To account for differences in the sequencing runs, the reads were initially downsampled so that the number of gigabases were equivalent in both the irradiated and non-irradiated samples' fastq files and the resulting non-irradiated sample telomere reads were downsampled to 14252 to closely match the 14254 telomere reads in the irradiated sample. Lastly, the chromosome ends were downsampled to 35 reads per chromosome end in order to produce Figure S3. The motif finding algorithm HOMER was used to determine telomere motifs by analyzing 1.5kb ends on each chromosome (Heinz et al., 2010). The discovered motifs were used to pull down all reads which contained any of these motifs repeated twice consecutively. The telomere reads from each respective sample were then split into 200bp windows with a 100 bp step.

Assessing telomere reads

Once each read containing the canonical repeats was extracted and split, the telomeric content was assessed. This was done with a custom Bash (Foundation, 2007) script which, due to the degenerate nature of *P. falciparum* telomeres, used much more promiscuous search patterns as opposed to the previously known telomere motifs. These patterns contained high GC content or high T content. The percent hit of the pattern was calculated across each 200bp sliding window. The telomere sliding window

data were then imported into multiple custom R (Team, 2013) scripts which determined the length and the distribution of telomere lengths, and assigned reads to each chromosome end based on the long-read mapping algorithm minimap2 (Li, 2018). Figures were then produced using ggplot2 (Wickham, 2016) in R (Team, 2013) and the python packages statannot (Weber, 2019) and seaborn (https://seaborn.pydata.org/). The statistics for the violin plots were produced using Welch's independent t-test for normally distributed data.

Supplemental References

Calhoun, S.F., Reed, J., Alexander, N., Mason, C.E., Deitsch, K.W., and Kirkman, L.A. (2017). Chromosome End Repair and Genome Stability in Plasmodium falciparum. MBio *8*.

Foundation, F.S. (2007). Bash (5.0)

Heinz, S., Benner, C., Spann, N., Bertolino, E., Lin, Y.C., Laslo, P., Cheng, J.X., Murre, C., Singh, H., and Glass, C.K. (2010). Simple combinations of lineage-determining transcription factors prime cis-regulatory elements required for macrophage and B cell identities. Mol Cell *38*, 576-589.

Li, H. (2018). Minimap2: pairwise alignment for nucleotide sequences. Bioinformatics *34*, 3094-3100.

Mattei, D., and Scherf, A. (1994). Subtelomeric chromosome instability in Plasmodium falciparum: short telomere-like sequence motifs found frequently at healed chromosome breakpoints. Mutat Res *324*, 115-120.

Salanti, A., Staalsoe, T., Lavstsen, T., Jensen, A.T.R., Sowa, M.P.K., Arnot, D.E., Hviid, L., and Theander, T.G. (2003). Selective upregulation of a single distinctly structured var gene in chondroitin sulphate A-adhering Plasmodium falciparum involved in pregnancy-associated malaria. Molecular Microbiology *49*, 179-191.

Team, R.C. (2013). R: A language and environment for statistical computing (Vienna, Austria: R Foundation for Statistical Computing).

Vembar, S.S., Seetin, M., Lambert, C., Nattestad, M., Schatz, M.C., Baybayan, P., Scherf, A., and Smith, M.L. (2016). Complete telomere-to-telomere de novo assembly of the Plasmodium falciparum genome through long-read (>11 kb), single molecule, real-time sequencing. DNA Res *23*, 339-351.

Weber, M. (2019). webermarcolivier/statannot.

Wickham, H. (2016). ggplot2: Elegant Graphics for Data Analysis (Springer-Verlag New York).
